# Plasma Proteins as Occupational Hazard Risk Monitors for Populations Working in Harsh Environments: A Mendelian Randomization Study

**DOI:** 10.3389/fpubh.2022.852572

**Published:** 2022-05-06

**Authors:** Ang Li, Wenjing Liao, Junyang Xie, Lijuan Song, Xiaowen Zhang

**Affiliations:** ^1^Department of Otolaryngology-Head and Neck Surgery, First Affiliated Hospital of Guangzhou Medical University, Guangzhou, China; ^2^State Key Laboratory of Respiratory Disease, Guangzhou, China

**Keywords:** workplace environment, plasma protein, occupational hazards, biomarker, Mendelian randomization

## Abstract

Harsh work environments can include very cold, hot, dusty, and noisy workplaces, as well as exposure in the workplace with chemicals and other fumes, cigarette smoke, and diesel exhaust. Although working in these harsh environments can have a negative effect on health, there are no effective biomarkers for monitoring health conditions until workers develop disease symptoms. Plasma protein concentrations, which reflect metabolism and immune status, have great potential as biomarkers for various health conditions. Using a Mendelian-randomization (MR) design, this study analyzed the effects of these harsh environments on plasma proteins to identify proteins that can be used as biomarkers of health status. Preliminary analysis using inverse variance weighted (IVW) method with a *p*-value cutoff of 0.05 showed that workplace environments could affect the concentrations of hundreds of plasma proteins. After filtering for sensitivity via MR-Egger, and Weighted Median MR approaches, 28 plasma proteins altered by workplace environments were identified. Further MR analysis showed that 20 of these plasma proteins, including UNC5D, IGFBP1, SCG3, ST3GAL6, and ST3GAL2 are affected by noisy workplace environments; TFF1, RBM39, ACYP2, STAT3, GRB2, CXCL1, EIF1AD, CSNK1G2, and CRKL that are affected by chemical fumes; ADCYAP1, NRSN1, TMEM132A, and CA10 that are affected by passive smoking; LILRB2, and TENM4 that are affected by diesel exhaust, are associated with the risk of at least one disease. These proteins have the potential to serve as biomarkers to monitor the occupational hazards risk of workers working in corresponding environments. These findings also provide clues to study the biological mechanisms of occupational hazards.

## Introduction

Occupational exposures to hazardous conditions are important risk factors for non-communicable diseases, including cardiovascular disease, hearing disorders, respiratory disorders, and cancers (1, 2). Although the development of workplace protection devices, such as protective clothing and masks, has dramatically reduced environmentally associated occupational hazards, individuals working in particular environments, such as coal mines and steel mills, are exposed to the health conditions that are less than optimal (3, 4). Moreover, no biomarkers have yet been identified to efficiently evaluate the health conditions of those workers prior to their development of diseases with obvious symptoms.

Proteins in the circulatory system are indicative of the physiology of individuals. Plasma protein level reflects allele differences, inflammatory status, and metabolic status (5). The variations of plasma protein levels in a number of diseases like hepatic and renal disease, acute myocardial infarction, cancer, diabetes, hyperlipidemia, and inflammatory diseases have been reported decades ago (6). More than 100 plasma proteins are cleared or approved by US Food and Drug Administration (FDA) as biomarkers clinically (e.g., C-reactive protein increase is used to predict coronary disease risk; thyroglobulin level is used to detect the recurrence of metastatic thyroid cancer after thyroid removal) and more and more plasma protein biomarkers are developed (5, 7, 8). Identifying plasma biomarkers that can monitor the health status of individuals working in extreme environments would be advantageous. Although the technology of plasma proteome profiling is rapidly developing (9), profile plasma proteins for a large cohort of workers working in a poor working environment, however, would be both costly and labor-intensive.

Mendelian randomization (MR) is a genetic epidemiological method that use genetic variants as instrumental variables to assess the causal relationship between exposures and outcomes (10). MR analysis has been used to investigate the causal association between fibroblast growth factor 23 level and bone mineral density of heel and femoral neck (11). The causal associations between educational attainment and 14 urological and reproductive health outcomes are also assessed via MR analysis (12). In this study, we performed two-sample MR to screen and evaluate the relationships between working in harmful environments (including very cold, hot, dusty, and noisy workplaces, as well as exposure in the workplace with chemicals and other fumes, cigarette smoke, and diesel exhaust, based on workers' self-reports) and the plasma concentrations of 2,992 proteins in European population. This study also assessed the relationships between plasma proteins potentially affected by workplace environments and the risks of disease by screening all 182 diseases related Genome Wide Association Study (GWAS) datesets collected by MRBASE (13) to estimate whether the variant of these plasma protein levels have potential effect on disease risks.

## Methods

### Data Sources of Genetic Associations With Workplace Environments

The UK Biobank (UKB) cohort collected and generated genetic and health information on more than half a million individuals in the UK participants (14). GWAS data of subjects working in very noisy workplaces (Self-reported: Often, *n* = 17,469, ncontrol = 73,184), very cold workplaces (Self-reported: Often, *n* = 7,881, ncontrol = 82,307), very hot workplaces (Self-reported: Often, *n* = 10,169, ncontrol = 79,996), very dusty workplaces (Self-reported: Often, *n* = 9,561, ncontrol = ), workplace full of chemical or other fumes (Self-reported: Often, *n* = 5,872, ncontrol = 82,863), workplace had a lot of second-hand cigarette smoke (Self-reported: Often, *n* = 14,941, ncontrol = 74,862), workplace had a lot of diesel exhaust (Self-reported: Often, *n* = 3,483, ncontrol = 85,621) were extracted from the second round GWAS results of the UK Biobank released in August 2018 (http://www.nealelab.is/uk-biobank). The summarized data used in this study were from the MRBASE (http://app.mrbase.org/) collection (13). Detailed GWAS ID and description of these data in MRBASE are shown in Supplementary Data 1.

### Data Sources of Genetic Associations With Plasma Protein Concentrations

GWAS analyses of the concentrations of 2,994 plasma proteins in 3,301 individuals of European descent have been reported (15). The GWAS summaries of the concentrations of 2,992 non-redundant plasma proteins collected by the MRBASE were used in this study as outcomes (Supplementary Data 1).

### Data Sources of Genetic Associations With Diseases

To investigate the role of plasma proteins detected in the preliminary analysis, all 182 disease related GWAS datasets of European population collected by MRBASE are used as outcomes to perform the second round of MR analysis. Detailed information about these GWAS datasets are included in Supplementary Data 1.

### Selection of Genetic Instruments and Statistical Analysis

**Figure 1:**
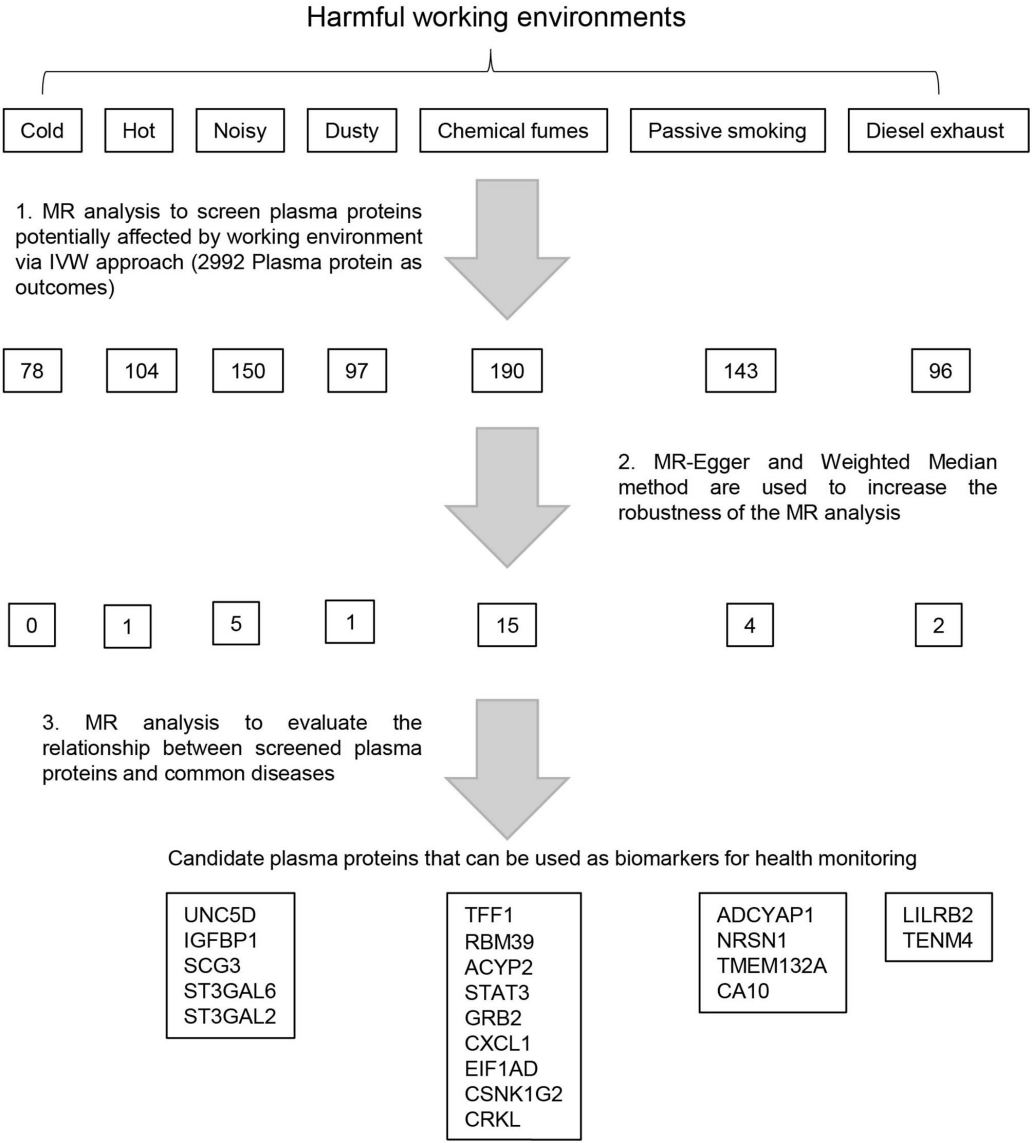
Framework of the study design. Preliminary Mendelian randomization (MR) analysis was performed using Genome Wide Association Study (GWAS) summary data of seven types of harmful working environments as exposures and GWAS summary data of 2,992 plasma proteins as outcomes. The robustness of the MR analysis was subsequently increased using MR-Egger and Weighted Median methods. The GWAS summary data for the selected plasma proteins are then used as exposures to evaluate the relationships between the plasma concentrations of these proteins and the risks of 28 diseases. Plasma proteins associated with the risk of at least one disease were selected as biomarkers to monitor corresponding occupational hazards.

Single nucleotide polymorphisms (SNPs) that strongly and independently predicted exposures of genome-wide significance were extracted using the extract_instruments function in TwoSanpleMR package (V 0.5.5) (13) with default parameters except for an alternative significance threshold (*P* < 5 × 10^−6^). Preliminary relationships between exposures and outcomes were estimated using the inverse variance weighted (IVW) approach (16). Results significant on IVW analysis were subjected to sensitivity analyses with different assumptions, including MR-Egger and weighted median (17, 18). To evaluate the relationships between the concentrations of identified plasma proteins and working environment related disease, the second round of MR analysis was performed using the IVW method, with plasma protein concentration as exposure and disease as outcome. The workflow of this analysis is shown in Figure 1.

All statistical analyses were performed using R version 4.0.3 with the TwoSampleMR package 0.5.5 (13). All summary data used are publicly available, so approval by an ethics committee was not required.

## Results

### Screening of Plasma Proteins Potentially Affected by the Workplace Environment

**Figure 2:**
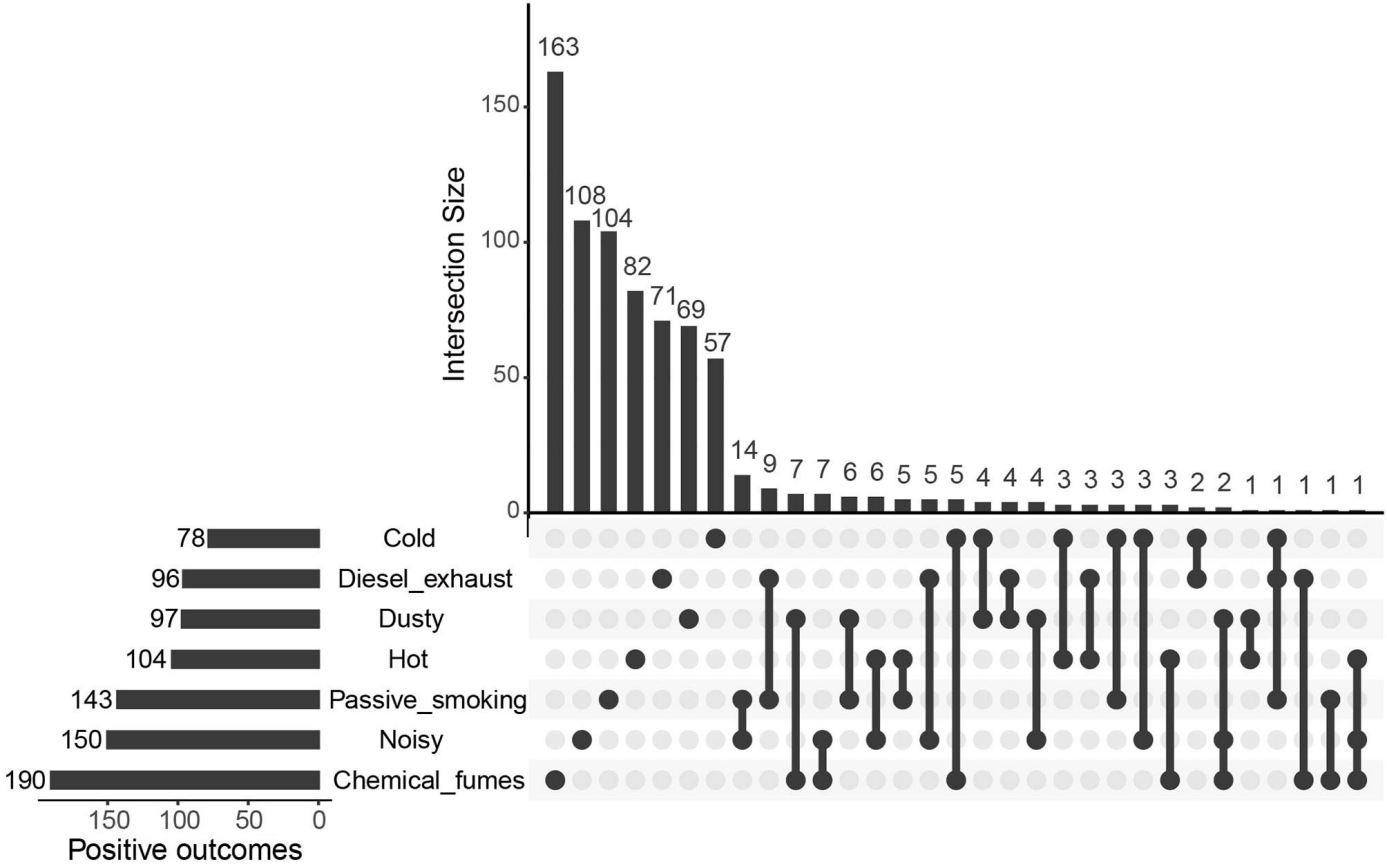
Preliminary screening of plasma proteins possibly affected by workplace environments. Mendelian randomization (MR) results with *p* < 0.05 were counted, with the Upset Plot showing outcomes overlapping among exposures to different environments.

Preliminary screening of seven workplace environments and 2,992 plasma protein levels using the IVW approach revealed that hundreds of plasma proteins were significantly associated with workplace environments (*P* < 0.05), including 78, 96, 97, 104, 143, 150, and 190 proteins significantly associated with exposure to cold, diesel exhaust, dust, hot, passive smoking, noise and chemical fumes, respectively (Figure 2 and Supplementary Table 1). The plasma proteins affected by different environmental factors showed limited overlap (Figure 2).

To increase the sensitivity of the MR analysis, MR-Egger, and Weighted Median approaches were performed. Twenty-eight plasma proteins (Table 1) passed the significance thresholds for all three method (*P* < 0.05). No plasma protein was robustly affected by a cold workplace environment. A noisy working environment was associated with a significant increase in plasma concentrations of CMP-N-acetylneuraminate-beta-galactosamide-alpha-2,3-sialyltransferase 2 (ST3GAL2) and significant decreases in concentrations of insulin-like growth factor-binding protein 1 (IGFBP1), secretogranin-3 (SCG3), type 2 lactosamine alpha-2,3-sialyltransferase (ST3GAL6), and unc-5 netrin receptor D (UNC5D) levels. A hot working environment was associated with a significant decrease in plasma collagen alpha-1 (COL1A1) concentration, whereas a dusty environment was associated with an increase in gap junction alpha-8 protein (GJA8). Exposure to chemical or other fumes altered the levels of 15 plasma proteins. Thirteen were up-regulated, including FAM107B, tyrosine kinase Fyn (FYN), growth factor receptor-bound protein 2 (GRB2), heparan sulfate glucosamine 3-O-sulfotransferase 3A1 (HS3ST3A1), bifunctional 3'-phosphoadenosine 5'-phosphosulfate synthase 1 (PAPSS1), acylphosphatase-2 (ACYP2), RNA-binding protein 39 (RBM39), 40S ribosomal protein SA (RPSA), signal transducer and activator of transcription 3 (STAT3), probable RNA-binding protein EIF1AD (EIF1AD), Crk-like protein (CRKL), and casein kinase I isoform gamma-2 (CSNK1G2). Exposure to chemical fumes reduced the plasma concentrations of growth-regulated alpha protein (CXCL1) and ephrin-A3 (EFNA3) and tended to decrease the concentration of trefoil factor 1 (TFF1). Passive smoking increased the levels of transmembrane protein 132A (TMEM132A) and carbonic anhydrase-related protein 10 (CA10) while decreasing the levels of neurensin-1 (NRSN1) and pituitary adenylate cyclase-activating polypeptide 38 (ADCYAP1). Diesel exhaust altered the plasma concentrations of leukocyte immunoglobulin-like receptor subfamily B member 2 (LILRB2) and teneurin-4 (TENM4). These findings suggest that these 28 plasma proteins were candidate biomarkers to monitor the health conditions of workers working in the corresponding environments.

**Table 1:** Results of Mendelian randomization (MR) analysis of the associations between workplace environments and plasma protein levels using three MR methods.

**Exposures**	**SNPs**	**IVW**		**MR Egger**		**Weighted median**	
**Outcomes**		**β (se)**	**pval**	**β (se)**	**pval**	**β (se)**	**pval**
**Noisy**
IGFBP1	18	−0.309 (0.102)	0.002	−0.73 (0.286)	0.021	−0.429 (0.139)	0.002
SCG3	18	−0.285 (0.128)	0.026	−0.92 (0.328)	0.013	−0.307 (0.156)	0.048
ST3GAL2	18	0.229 (0.108)	0.034	0.806 (0.286)	0.012	0.338 (0.144)	0.019
ST3GAL6	18	−0.329 (0.102)	0.001	−0.805 (0.286)	0.012	−0.328 (0.142)	0.021
UNC5D	18	−0.316 (0.125)	0.011	−0.751 (0.341)	0.043	−0.342 (0.147)	0.020
**Hot**
COL1A1	13	−0.277 (0.095)	0.003	−0.532 (0.21)	0.028	−0.364 (0.132)	0.006
**Dusty**
GJA8	15	0.198 (0.089)	0.026	0.527 (0.22)	0.032	0.228 (0.114)	0.045
**Chemical fumes**
ACYP2	20	0.178 (0.065)	0.006	0.45 (0.127)	0.002	0.186 (0.085)	0.029
CRKL	20	0.134 (0.061)	0.028	0.272 (0.127)	0.046	0.186 (0.08)	0.019
CSNK1G2	20	0.138 (0.061)	0.023	0.284 (0.127)	0.038	0.179 (0.082)	0.028
CXCL1	20	0.148 (0.073)	0.042	0.43 (0.137)	0.006	0.171 (0.082)	0.038
EFNA3	20	−0.159 (0.066)	0.017	−0.311 (0.136)	0.035	−0.165 (0.081)	0.042
EIF1AD	20	0.179 (0.07)	0.010	0.363 (0.141)	0.019	0.187 (0.085)	0.028
FAM107B	20	0.217 (0.061)	0.000	0.428 (0.127)	0.003	0.201 (0.084)	0.017
FYN	20	0.192 (0.062)	0.002	0.505 (0.127)	0.001	0.168 (0.085)	0.048
GRB2	20	0.151 (0.061)	0.013	0.284 (0.127)	0.038	0.189 (0.086)	0.028
HS3ST3A1	20	0.166 (0.061)	0.006	0.275 (0.127)	0.043	0.178 (0.086)	0.039
PAPSS1	20	0.121 (0.061)	0.046	0.325 (0.127)	0.019	0.169 (0.084)	0.043
RBM39	20	0.209 (0.061)	0.001	0.407 (0.127)	0.005	0.224 (0.081)	0.006
RPSA	20	0.18 (0.061)	0.003	0.294 (0.127)	0.032	0.18 (0.083)	0.031
STAT3	20	0.168 (0.061)	0.006	0.311 (0.127)	0.024	0.161 (0.082)	0.049
TFF1	20	−0.148 (0.068)	0.029	−0.468 (0.127)	0.002	−0.222 (0.085)	0.009
**Passive smoking**
ADCYAP1	18	−0.275 (0.096)	0.004	−0.534 (0.23)	0.034	−0.284 (0.132)	0.031
CA10	18	0.417 (0.106)	0.000	0.604 (0.255)	0.031	0.546 (0.142)	0.000
NRSN1	18	−0.26 (0.112)	0.020	−0.56 (0.262)	0.048	−0.299 (0.132)	0.023
TMEM132A	18	0.252 (0.096)	0.009	0.552 (0.229)	0.029	0.291 (0.133)	0.029
**Diesel exhaust**
LILRB2	22	−0.145 (0.044)	0.001	−0.237 (0.091)	0.017	−0.165 (0.06)	0.006
TENM4	22	0.143 (0.044)	0.001	0.221 (0.091)	0.025	0.159 (0.058)	0.006

### Plasma Proteins as Biomarkers for Health Monitoring

To evaluate the associations of the 28 selected plasma proteins with the risk of diseases, MR analyses of the concentrations of these 28 plasma protein and 182 diseases were performed. COL1A1 and GJA8, which were found to be affected by hot and dusty environments, respectively, were unrelated to the diseases tested (*p* > 0.145 and *p* > 0.074, respectively).

**Figure 3:**
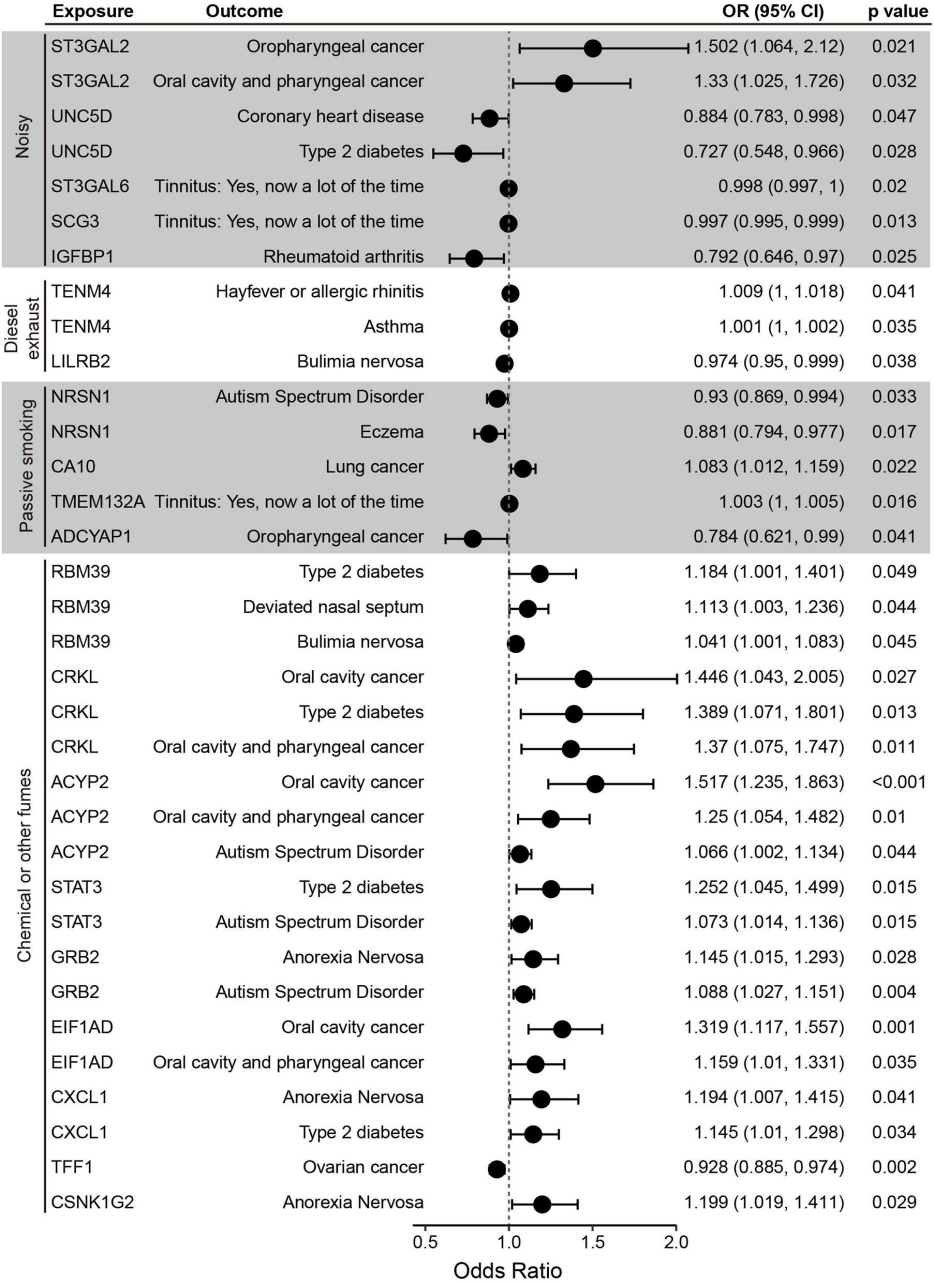
Forest plot of Mendelian randomization (MR) results of the relationships between plasma proteins and selected diseases. Shown are the odds ratios (ORs), 95% confidence intervals (CIs) and *p*-values of MR analysis performed using the inverse variance weighted (IVW) method.

Five plasma proteins affected by noisy environments were associated with at least one of the diseases tested (Figure 3). MR analysis showed that ST3GAL2, which is elevated in noisy workplaces, is a risk factor for oropharyngeal, oral cavity, and pharyngeal cancer. In contrast, UNC5D, ST3GAL6, SCG3, and IGFBP1, which are negatively affected by noisy workplace environments, are potentially protective factors against coronary heart disease (UNC5D), type 2 diabetes (UNC5D), tinnitus (ST3GAL6, SCG3), and rheumatoid arthritis (IGFBP1).

Exposure to diesel exhaust fumes increased the plasma concentration of TENM4, a protein involved in neural development (19) and a risk factor for hayfever, allergic rhinitis and asthma (Figure 3). Exposure to diesel exhaust was found to decrease plasma levels of LILRB2, a potentially protective factor against bulimia nervosa (Table 1 and Figure 3).

NRSN1 and ADCYAP1 were found to be negatively affected by passive smoking (Table 1). MR analysis showed that NRSN1 is a protective factor against autism spectrum disorders and eczema, and that ADCYAP1 is protective against oropharyngeal cancer (Figure 3). CA10 and TMEM132, which were upregulated by passive smoking, are predicted risk factors for lung cancer and tinnitus, respectively (Figure 3).

Nine of the fifteen plasma proteins affected by chemical or other fumes were associated with disease risk (Figure 3). RBM39, CRKL, ACYP2, STAT3, GRB2, EIF1AD, and CXCL1 are risk factors for multiple diseases, including type 2 diabetes, oral cavity and pharyngeal cancer, deviated nasal septum, bulimia nervosa, anorexia nervosa, and autism spectrum disorder, and three proteins, GRB2, CXCL1, and CSNK1G2, are risk factors for anorexia nervosa. TFF1, which is decreased by chemical fumes, is a protective factor against ovarian cancer.

In summary, these 20 plasma proteins were found to be associated with workplace environments and disease. These candidate of biomarkers may be useful for monitoring the health condition of workers.

## Discussion

This study evaluated the roles of the workplace environment on plasma protein levels. These analyses showed that distinct plasma proteins are affected by different environments. Cold, hot, and dusty environments had less of an effect on plasma protein levels than noise, chemical fumes, passive smoking, and diesel exhaust.

COL1A1 is likely to be decreased when working in hot environment and GJA8 is likely to be increased when working in dusty environments based on present study. COL1A1 encodes the pro-alpha1 chain of type I collagen, a fibril-forming collagen found in most connective tissues. COL1A1 may be a biomarker and therapeutic target for multiple cancers (20, 21). A recent study showed that a plasma COL1A1 concentration >256.5 ng/mL was associated with poor survival from heart failure in heart transplant recipients (22). The decrease in plasma COL1A1 induced by hot workplace environments may positively affect health. GJA8 is a connexin protein necessary for lens growth, with mutations in the gene encoding this protein associated with multiple eye diseases (23, 24). There is no evidence that an increased GJA8 level has adverse effects.

Thermoregulatory systems in mammals are robust and reliable after millions of years of evolution. Although cold (winter season) has been reported to increase cardiovascular disease morbidity and mortality rates (25, 26), the effect of exposure to cold is unclear. The causes of these increases in morbidity and mortality are complex, and the winter season cannot fully represent exposure to cold. Exposure to cold has been found to activate brown adipose tissue (BAT), which is believed helpful for physical fitness (27–30). Sauna bathing is a type of exposure to heat associated with a reduced risk of sudden cardiac death (31). However, sauna bathing is always coupled with cooling periods in cold environments, suggesting the need for studies of physiological adaptations during the shift from hot to cold environments (29). Based on these findings and the results of the present study, temperature in the workplace is not likely to affect workers' health wearing proper protection. Dusty environments have been associated with respiratory diseases in the absence of proper protection (32). Dust particles, however, are rather large, with most face masks preventing exposure to dust. Indeed, the incidence of pneumoconiosis has decreased in recent years (33).

The present study found that exposure to noise, diesel exhaust fumes, second-hand cigarette smoke, and chemical or other fumes have distinct effects on plasma protein concentrations. Further MR analysis of the plasma proteins affected by workplace environments and disease risks have shown results consistent with previous findings. For example, many studies have shown that overexposure to noise is associated with tinnitus (34, 35). The present study showed that noisy workplaces decreased plasma concentrations of ST3GAL6 and SCG3, proteins thought to protect against tinnitus. This study also showed that workplace exposure to second-hand smoke was associated with increased plasma levels of CA10, a risk factor for lung cancer. These results suggest that the relationships between workplace environments and the identified plasma proteins were both reliable and significant.

MR analyses in the present study have found associations of environmentally affected plasma proteins with oral cavity and oropharyngeal cancers. Household air pollution from the utilization of solid fuels was found to be an independent risk factor for oral cancer (36), and exposure to petroleum-based and oxygenated solvents was associated with the risks of oral and oropharyngeal cancers, although no exposure-response trend was observed (37). The findings in the present study are consistent with these earlier results, providing new evidence for the association between environmental pollution and oral and oropharyngeal cancers.

Most plasma proteins identified in this study are not secreted proteins. They most likely come from exosomes secreted by cells in a particular tissue or organ. Although MR analysis is robust and reliable, further study is needed to validate these biomarkers. The function of these proteins in particular occupational hazards is also worth further investigation.

## Conclusions

This study suggested that harsh environments may affect the concentrations of multiple plasma proteins and the change of those plasma proteins concentration have great potential to be used as biomarkers to predict occupational hazards risk. Twenty plasma proteins are recommended as biomarkers to monitor the health condition of workers working in noisy workplaces, workplaces full of diesel exhaust, workplaces full of smoke from other smokers, and workplaces full of chemical or other fumes. More epidemiologic studies are needed to test the sensitivity, specificity, and overall accuracy of these candidate biomarkers.

## Data Availability Statement

The source data for Figure 2 are included in Supplementary Data 2. The source data for Figure 3 are included in Supplementary Data 3. Further inquiries can be directed to the corresponding authors.

## Ethics Statement

Ethical review and approval was not required for the study on human participants in accordance with the local legislation and institutional requirements. Written informed consent for participation was not required for this study in accordance with the national legislation and the institutional requirements.

## Author Contributions

AL: conceptualization, methodology, software, and writing—original draft preparation. WL, JX, and LS: writing—reviewing and editing. XZ: supervision. All authors contributed to the article and approved the submitted version.

## Conflict of Interest

The authors declare that the research was conducted in the absence of any commercial or financial relationships that could be construed as a potential conflict of interest.

## Publisher's Note

All claims expressed in this article are solely those of the authors and do not necessarily represent those of their affiliated organizations, or those of the publisher, the editors and the reviewers. Any product that may be evaluated in this article, or claim that may be made by its manufacturer, is not guaranteed or endorsed by the publisher.

## Supplementary Material

Supplementary Table 1 Number of plasma proteins showed association with workplace environments in preliminary Mendelian randomization (MR) analysis via inverse variance weighted (IVW) approach.

Supplementary Data 1 GWAS datasets used for MR analysis in this study.

Supplementary Data 2 MR results between workplace environments and plasma proteins.

Supplementary Data 3 MR results between plasma proteins and disease risk.
