# Supplementary material for: Plasma Proteins as Occupational Hazard Risk Monitors for Populations Working in Harsh Environments: A Mendelian Randomization Study
Source: Front Public Health. 2022 May 6;10:852572. doi: 10.3389/fpubh.2022.852572 (PMC9120921; doi:10.3389/fpubh.2022.852572)
Supplement: Supplementary Table 1 — Number of plasma proteins showed association with workplace environments in preliminary Mendelian randomization (MR) analysis via inverse variance weighted (IVW) approach. [file Table_1.pdf]

Table S1. Number of plasma proteins showed association with workplace environments in preliminary Mendelian randomization (MR) analysis via inverse variance weighted (IVW) approach.

Workplace Environments	Associated plasma proteins (p<0.05)	Other plasma proteins (p>=0.05)	Total
Cold	78	2916	2994
Diesel exhaust	96	2898	2994
Dust	97	2897	2994
Hot	104	2890	2994
Passive smoking	143	2851	2994
Noise	150	2844	2994
Chemical fumes	190	2804	2994
